# Working with parents to counteract bullying: A randomized controlled trial of an intervention to improve parent‐school cooperation

**DOI:** 10.1111/sjop.12522

**Published:** 2019-02-12

**Authors:** Coby van Niejenhuis, Gijs Huitsing, René Veenstra

**Affiliations:** ^1^ Department of Sociology University of Groningen Groningen The Netherlands

**Keywords:** Bullying, parental involvement, parents, school, teachers

## Abstract

This study examined the effectiveness of an intervention aimed at improving parent‐school cooperation in counteracting bullying. Using a randomized controlled trial, data of teachers, parents of non‐victimized children, and children themselves were collected at 13 intervention and 14 control schools (grades 3–6, *N at post‐assessment:* teachers = 83, parents = 153, children = 2,510) at two time points (time lag about 6 months). Results showed positive effects of the intervention for some aspects of the primary outcomes: parents’ and teachers’ attitudes and efforts, whereas no effects were found of teachers’ or parents’ competences in counteracting bullying. No intervention effects were found for secondary outcomes: children's self‐reported bullying, victimization, well‐being, and self‐esteem. The findings indicate that, due to the intervention, teachers and parents were more aligned and able to cooperate, even within the short time of the intervention: one school year. This is the first essential step to systematically addressing parents’ role in tackling bullying; future research is needed to examine the long‐term effects of parent and school interventions in enhancing the effectiveness of anti‐bullying programs.

## Introduction

Bullying is goal‐directed, repeated, and intentional hurting of children within the context of a power imbalance implying that victims cannot defend themselves (Olweus, 1993; Volk, Dane & Marini, 2014). Bullying is a common and universal problem (Craig, Harel‐Fisch, Fogel‐Grinvald, Dostaler, Hetland & Simons‐Morton, 2009), and has negative consequences for everyone involved. For example, victims of bullying are at risk of long‐term internalizing problems, such as low self‐esteem, depressive symptoms, and anxious feelings (Arseneault, 2018; Hawker & Boulton, 2000; Reijntjes, Kamphuis, Prinzie & Telch, 2010). Bullies are at risk of externalizing problems, such as delinquent behavior and substance abuse later in life (Kretschmer, Veenstra, Branje *et al*., 2018; Ttofi, Farrington, Loesel & Loeber, 2011). Bystanders, who are not actively involved in bullying are often afraid of becoming victims themselves and feel unhappy because they often want to intervene but do not dare to do so (Nishina & Juvonen, 2005; Rivers, Poteat, Noret & Ashurst, 2009). For these reasons, it is of vital importance to design effective interventions against bullying.

Most bullying takes place at school; therefore, schools should take the lead in counteracting bullying. Teachers have an important role in steering classroom group processes and in clearly disapproving of bullying (Veenstra, Lindenberg, Huitsing, Sainio & Salmivalli, 2014). More and more schools are taking responsibility for tackling bullying, whether or not pressured by national laws. However, schools cannot do this alone. Parents also have an important role in counteracting bullying. Parents are among the most important people in a child's life (Von Salisch, 2001). They know their child the longest and, in many ways, the best. Consequently, they usually know what approaches will be successful or not for their child. Moreover, there is a high degree of loyalty within the parent‐child relationship, which means that parents have a strong influence on their child. Therefore, schools and parents need each other to prevent, signal, and tackle bullying. For this reason, we developed an additional intervention component for anti‐bullying programs that was specifically aimed at counteracting bullying through parent‐school cooperation. In the current study, we evaluated this supplementary intervention component.

### Parent‐school cooperation in counteracting bullying

It is important for the social development of children in general and for the prevention of bullying behavior in particular that school and parents convey the same message to children (Ostrander, Melville, Bryan & Letendre, 2018; Sheridan, Erchul, Brown *et al*., 2004). School and parents should try to *prevent* bullying by unanimously speaking out against it and by agreeing on how bullying should be *tackled*. Moreover, it is important that parents and schools exchange *signals* of bullying to prevent (further) negative consequences of bullying.

Even though elementary school teachers are supposed to have a central role in tackling bullying, many do not recognize the victims in their classrooms (Oldenburg, Bosman & Veenstra, 2016). Research among primary school children shows that only 53% of the bullied children reported bullying to the teacher, whereas 67% told their parents (Fekkes, Pijpers & Verloove‐Vanhorick, 2005). Thus, children generally tell their parents more easily that they are being bullied than they tell teachers (see also: Whitney & Smith, 1993).

Schools and parents can mutually benefit from information exchange to signal bullying, because there will always be children who tell at school but not at home about their victimization, and vice versa. This mutual dependence also applies to recognizing bullies; parents are often unaware of the bullying behavior of their children (Holt, Kaufman Kantor & Finkelhor, 2009).

That children hide bullying behavior from parents or teachers is not surprising, because bullying behavior is usually rejected. That victims do not reveal their situation to parents or teachers, however, does not speak for itself. Possible reasons are that children often think that the bullying is not serious enough, that adults cannot solve the problem, or that they are afraid of retaliation by the bullies (Newman & Murray, 2005). These expectations may be realistic, as research indicates that many parents who are aware of bullying do not take any action (Holt *et al*., 2009; Lovegrove, Bellmore, Green, Jens & Ostrov, 2013). Moreover, when parents do take action, this generally does not result in a decrease in bullying; for a small group of children it even leads to increases in bullying. Thus, similar to the *prevention* and *signaling* of bullying, it is important for *tackling* bullying that consistent and constructive actions are taken by both schools and parents. Cooperation between the two is essential for improving the situation (Harcourt, Jasperse & Green, 2014). Many schools acknowledge the importance of cooperation with parents, but often lack ideas about how they can effectively involve parents in counteracting bullying. Anti‐bullying programs are often complemented with materials to help schools involve parents. An analysis of the contents of 44 anti‐bullying programs revealed that an important element of successful anti‐bullying programs is parental involvement through parent training or meetings (Farrington & Ttofi, 2009). Elaborating on this, we developed a substantive intervention to enhance parental involvement in schools, with a specific focus on counteracting bullying. The intervention is an add‐on for existing anti‐bullying programs, but it can also be used by primary schools that do not use an anti‐bullying program.

### The intervention

The intervention ‘Working with parents in creating a pleasant school’ consists of teacher materials (toolkit) and a training course. The intervention targets teachers, who should function as independent professionals. In the training sessions, teachers are equipped with tools to foster cooperation with parents. Thus, teachers can involve parents in the school without depending on external persons, such as the trainers of the intervention.

The intervention was based on four insights that schools should address to create and maintain parental involvement in counteracting bullying. First, schools should provide a clear vision on parent‐school cooperation, and translate this vision into school policies and practices (Hawley & Williford, 2015). It must be clear to parents and all school personnel where the school stands on counteracting bullying, parental involvement, and the combination of these. A clearly formulated vision is essential for the team members to know what they can expect of each other, and for parents to know what they can expect of the team (Smit, Sluiter & Driesen, 2006; Van Loo, 2014). This vision is the foundation from which the school starts parent‐school cooperation.

Second, schools should enable parents to form a group. This is essential for social capital to produce mutual trust, norms, and reciprocity (Bourdieu, 1986; Putnam, 1995). Parents will not only be involved with their own child, but also with other children in the school. Parents who feel that they are part of a community are more involved with the school as a whole (Barge & Loges, 2003; Henderson & Mapp, 2002). Moreover, parents are a role model for their children, and positive relations between parents are essential for positive contact between their children. The practical implication is that schools organize collective contact moments where parents can meet and exchange ideas.

Third, the bond between teachers and parents should be strengthened. Interaction moments between teachers and parents can be used to discuss children's feelings at school, their position in the group, and possible intervention strategies (if necessary). Frequent interaction is an essential basis for mutual trust and lowers the threshold for further contact (Deslandes & Bertrand, 2005). When teachers start sharing positive information with parents, this forms the basis for sharing negative content messages, such as children's involvement in bullying or victimization.

The fourth insight encourages structural written contact about efforts to create a safe and pleasant school atmosphere (e.g., news or information letters). Face‐to‐face contact with parents offers interaction opportunities, and is therefore preferable. However, regular letters (either on paper or digital) can also be efficient and effective to keep each other informed and to keep the mutual contact going.

Teachers at intervention schools participated in two separate training days per school, which each lasted four hours. Day one took place within 4 weeks after the school year started, and addressed several content‐related topics (see next subsection). The teachers prepared an activity to involve parents in counteracting bullying, which was carried out before the second training day. Day two took place about 6 weeks after the first session, and evaluated the activity that was implemented. The training course ended with the selection of parent‐school activities for the coming school year, the scheduling of these activities, and the assigning of responsible persons. During the implementation of the intervention, a toolkit was used containing activities, teaching methods, and additional suggestions that schools could use to enhance parent‐school cooperation in general, and specifically with respect to bullying.

### The present study

This study examined the effectiveness of the above‐mentioned intervention in improving parent‐school cooperation in counteracting bullying in a randomized controlled trial in Dutch primary schools (14 control and 13 intervention schools). The data were collected at two time points using online questionnaires (October and May of the same school year).

The intervention was primarily aimed at changing the *attitudes*,* efforts*, and degree of *competence* of both teachers and parents (e.g., Veenstra *et al*., 2014). The main aim of the first part of the training course was to give teachers insight into their current attitudes, efforts, and (perceived) competences. The key questions were: What are the teachers’ attitudes to bullying and parental involvement? Are the attitudes of all school staff aligned? To what extent can their attitudes be recognized in their daily (efforts)? Do they feel competent to involve parents in counteracting bullying? Using this information as baseline, the training course was aimed at (further) improving the attitudes, efforts, and competences of the teachers as a team. In doing so, the need to work on the four aforementioned insights was stressed. When implementing parental activities, teachers were expected to enhance the attitudes, efforts, and competences of the parents.

In line with our primary aim, we tested whether the intervention had an impact on the following outcomes: (1a) stronger anti‐bullying *attitudes* of both teachers and parents and (1b) a more positive attitude toward mutual cooperation in counteracting bullying for both teachers and parents; (2) the *efforts* of both teachers and parents in tackling bullying (together); (3) the *competences* of both teachers and parents to tackle bullying (together).

We expected that this universal intervention (i.e., aimed at all teachers and parents) would affect only the parents of non‐victimized children, because – compared with the parents of victimized children – they usually pay less attention to this topic. For example, the parents of non‐victimized children were expected to have less contact with both their child and the teachers about bullying issues, be less informed about bullying and its consequences, have a less clear‐cut attitude toward bullying, and perhaps miss future signals of bullying or victimization in their child. For that reason, the intervention was most likely to achieve a positive change in attitudes, efforts, and competences for these less‐involved parents. Parents who were aware of the victimization of their children were expected already to have intensive contact with the school and practice strategies to help their children (Malm, Henrich, Varjas & Meyers, 2017); they might therefore benefit more from a targeted intervention.

We also tested changes in *secondary* outcomes: whether the intervention (4a) reduced children's bullying and victimization and (4b) improved their well‐being at school and self‐esteem. We considered children's outcomes as secondary outcomes because the intervention was focused primarily on teachers: they followed the training and used the toolkit to involve parents more intensively in the school and, specifically, to counteract bullying. This should be noticed directly by the parents. Increased parent‐school cooperation may lead indirectly to changes in the children's behavior, which we may not find within the time frame of the school year in which the intervention is implemented.

## Method

### Participants and procedure

The intervention was used as an additional component in the KiVa anti‐bullying program (Salmivalli, Kärnä & Poskiparta, 2010) as this was implemented in the Netherlands (Kaufman, Kretschmer, Huitsing & Veenstra, 2018; Veenstra, 2015). To date, the KiVa program has included materials for a parental information evening (organized and delivered by schools) and an extensive parental brochure. In the supplementary intervention, we added the training course and toolkit to these materials. We compared teachers, parents, and children at KiVa schools that made use of the additional intervention with a control group of teachers, parents, and children at KiVa schools using the regular parental approach. Conducting the research on KiVa schools only has the advantage that possible intervention effects cannot be attributed to differences in anti‐bullying programs.

In the spring of 2015, all Dutch schools working with the KiVa anti‐bullying program received an email and a letter about participation in this study (*N *=* *170 at that time). Twenty‐eight of these schools indicated that they wished to participate in the research, and half of these were randomly assigned to the intervention condition. The intervention was delivered for free in the school year 2015–2016. The 14 control schools followed a “care as usual” approach to parental involvement, and were offered the possibility to implement the intervention a year later. A stratified randomization procedure was used, with schools being paired based on the year in which they started with KiVa, the number of children in the school, and the average scores on children's self‐reported bullying in October 2014 (from the KiVa monitor). For each pair of schools, one school was randomly assigned to the intervention group and the other to the control group.

Before it started, the study was registered at the Netherlands Trial Register (NTR number 5796). Data were collected from teachers, parents, and children. Schools asked all teachers and parents to complete the online questionnaire before (October 2015) and after (May 2016) the implementation of the intervention. Although all teachers and parents were allowed to respond, we used only the teacher and parent data of children from grades three to six (Dutch grades five to eight) because: (1) teachers from these grades already worked with the KiVa intervention (many teachers from lower grades did not); (2) parental involvement in the higher grades is less self‐evident than in the lower grades, because the parents of toddlers usually have more frequent teacher contact when they bring their child into the classroom. Therefore, we expected the intervention to make a difference in the highest grades. In addition (3) the child questionnaire was only presented to children in grades three to six.

The online child questionnaires were completed in October 2015 and May 2016 as part of the biannual KiVa monitor. Before the schools started using this monitor, permission forms were sent to the parents. Parents who wished to keep their children from participating were requested to return the form to school. Children who did not receive parental permission did not participate in our study.

For several reasons, schools, teachers, parents, or children dropped out. One intervention school did not participate at all in the pre‐ and post‐assessment because a newly assigned principal did not agree with participation. The parents and teachers of another intervention school did not participate in the post‐test because of social upheaval in the team caused by illness and personnel changes. Finally, the pre‐test data of children in one of the control schools could not be used in the analyses because they had not been stored properly. Depending on the responses per measurement wave, the data of 23–27 schools, 83–93 teachers, 153–191 parents, and 2,510– 2,529 children were used for the analyses.

### Primary outcome measures

Information about teachers was retrieved directly by asking teachers about themselves and indirectly by asking parents about the teacher. Likewise, information about parents was retrieved by asking parents as well as teachers. This multi‐informant method was used because the focus of the intervention was parent‐school cooperation, so it was necessary to investigate whether there was perceived improvement from both the parents’ and the teachers’ perspectives. The teacher and parent information on attitudes, efforts, and competences is described as measured directly using the questionnaires. Appendix 1 provides items and answer categories, and shows that indirect information from the other informant was measured using comparable items. Some items that were added at the post‐assessment are written in italics and marked with “(T2).” Unless stated otherwise, answers were given on a five‐point scale (1 = completely false‐5 = completely true).

#### Teacher information

Teachers’ *attitudes to (counteracting) bullying* (Appendix 1 Table A1) were assessed using two single items: “It is my responsibility to prevent bullying in my classroom” and “Everyone that works at this school thinks it is important to counteract bullying.”

Teachers’ *attitudes to parental involvement* (Appendix 1 Table A1) were assessed using six statements, like: “I think it is important to inform parents (1) how we can prevent bullying together or (2) when I think their child is being bullied” (Cronbach's alpha = 0.77). Additionally, teachers answered the statement, “Everyone that works at this school thinks it is important to work together with parents to counteract bullying.”

Teachers’ *efforts regarding bullying* (Appendix 1 Table A2)were assessed using three retrospective items: “How much did you do this school year to (1) prevent bullying; (2) signal bullying; and (3) tackle bullying the moment it occurred” (Cronbach's alpha = 0.92). Answers were given on a five‐point scale (1 = much less than the previous school year, 2 = less than the previous year, 3 = no difference, 4 = more than the previous school year, 5 = much more than the previous school year).

Teachers’ *efforts regarding parental involvement* (Appendix 1 Table A2) were assessed using six single items about general efforts (e.g., “I do my best to make parents feel welcome”; “I contact parents if their child is not doing well”) and three single items about more specific efforts (e.g., “During conversations we (also) discussed whether the child likes going to school”). All items were answered using a five‐point scale, except for the two items that were only measured at the post‐assessment. Answers ranged from (1) much less than the previous year to (5) much more than the previous year.

Teachers’ *competence in counteracting bullying* (Appendix 1 Table A3) was assessed using two items: “I know how to (1) prevent bullying; (2) intervene when bullying occurs” (Cronbach's alpha = 0.86). The competence of *involving parents* (Appendix 1 Table A3) was measured using “I think it is difficult to: (1) involve parents in school; (2) contact parents if their child is being bullied; and (3) contact parents if their child is bullying others” (Cronbach's alpha = 0.78).

#### Parent information

Parental *attitudes to (counteracting) bullying* (Appendix 1 Table A1) were measured using two single items: “I think it is wrong if my child assists in bullying” and “I think it is (also) my responsibility to prevent bullying in the classroom.”

Parental *attitudes to parental involvement* (Appendix 1, Table A1) were measured using five items (Cronbach's alpha = 0.62), e.g., “I think it is important the teacher contacts me: (1) about how to prevent bullying together; (2) when my child is being bullied.” Two additional retrospective items were included in the questionnaire (Cronbach's alpha = 0.88): namely, “This school year, I felt more involved in: (1) creating a pleasant atmosphere at school; and (2) counteracting bullying at school.”

Parental *efforts regarding bullying* (Appendix 1 Table A2) were assessed using four items: “I like talking to my child about: (1) whom he/she plays with at school; (2) what he/she learned at school; (3) whether he/she likes it at school; and (4) whether other children are nice to him/her” (Cronbach's alpha = 0.85). Parents were also asked “How often did you talk to your child about these topics?” Answers to this question varied from (1) much less than the previous school year to (5) much more than the previous school year.

Items on parental *efforts regarding parental involvement* (Appendix 1 Table A2) concerned their responsiveness to the efforts of the school. Three single items were used: “This school year, (1) to what extent did you respond to invitations from school to talk about your child?; (2) to what extent did you respond to invitations for meetings that other parents attended?; and (3) how often did you read school correspondence?” Parents answered on a five‐point scale (1 = much less than the previous school year to 5 = much more than the previous school year).

Parental *competences in counteracting bullying* (Appendix 1 Table A3) were measured using four items describing how much parents learned in that school year. Examples are: “This school year, I learned more about (1) what bullying is; (2) what the consequences of bullying are” (Cronbach's alpha = 0.94). Four additional items measured parents’ perceived difficulties in counteracting bullying, e.g.: “I think it is difficult to (1) prevent my child from being bullied”; (1) ‘prevent my child from bullying others” (Cronbach's alpha = 0.74).

### Secondary outcome measures

For the secondary outcomes a multi‐informant method was used; parents reported on their children in addition to children's self‐reports (see Appendix 2). The measures in the child and parent questionnaires were identical, with the exception of self‐esteem (see below).

#### Child information

*Bullying* and *victimization* were assessed using the global questions from the revised Olweus’ Bully/Victim questionnaire (1996). The children were given a definition of bullying and subsequently asked to respond: “How often have you been bullied at school in the past couple of months?” They answered on a five‐point scale (0 = not at all, 1 = once or twice, 2 = two or three times a month, 3 = about once a week, 4 = several times a week).

*Well‐being at school* was measured using seven items (Kärnä *et al*., 2011) that formed a reliable scale (Cronbach's alpha = 0.94). Students responded on a four‐point Likert‐type scale (1 = never, 4 = always) to items reflecting general liking of school (e.g., “I like it at school”) and feelings of safety (e.g., “I feel safe at school”).

*Self‐esteem* was measured using a five‐item scale derived from the Rosenberg self‐esteem scale (1965). Only the positively formulated items were used to make the questions applicable to this age group. Students responded on a five‐point Likert‐type scale (1 = never true, 5 = always true) to items such as “I feel that I have a number of good qualities” and “I feel that I am a person of worth, at least on an equal plane with others” that formed a reliable scale (Cronbach's alpha = 0.82). Parent‐reported self‐esteem was based on one item (“My child has a high self‐ esteem”) with answers on a four‐point scale (1 = never, 2 = sometimes, 3 = often, 4 = always; Robins, Hendin & Trzesniewski, 2001).

### Analytical strategy

The effects of the intervention were investigated by comparing outcome measures at the intervention and control schools. Because schools were randomly assigned to the control and intervention conditions, the outcomes at control schools could be used to determine the average outcome that intervention schools would have achieved if they had not participated in the intervention. The difference in outcomes between the intervention and control schools is known as the intention‐to‐treat (ITT) effect. Multilevel (regression) analyses were conducted, with the *intervention* effect predicting the outcome measures, while taking account of the clustering of teachers, parents, and children in the schools. The analyses were aimed at testing whether the intervention worked; and the findings do not provide insight into possible effective components of the implementation fidelity. Initial analyses of the pre‐test (Appendix 3) showed that intervention and control schools did not differ systematically on any outcome measure at baseline. Therefore, the effectiveness of the intervention could be determined for follow‐up scores without taking account of baseline scores.

Although we expected intervention effects only for the parents of non‐victims, we performed additional analyses separately for parents who thought that their child was often victimized versus parents who thought that their child was not being victimized. These analyses showed, as expected, only one intervention effect on parents with victimized children and multiple intervention effects on parents of non‐victimized children. Therefore, only the findings for the latter group are shown in the results section.

## Results

The results for the primary outcome measures are given in Tables 1, 2, 3. The first column in these tables gives the average outcome of the control group, and the second column shows the difference between the intervention group and the control group.

**Table 1:** Primary outcomes: attitudes at the post‐assessment

	Teachers as informant		Parents of non‐victims as informant	
	Mean control	Difference intervention	Mean control	Difference intervention
1. Attitude to bullying				
a. Teacher (school)				
Prevention of bullying is responsibility of teacher.	4.43	0.02 (0.18)	4.10	0.24 (0.14)
Attitude of school to counteracting bullying.	4.75	0.09 (0.17)	4.48	0.11 (0.09)
b. Parent				
Parent disapproves of bullying.	3.82	0.61 (0.22)**	4.75	−0.35 (0.15)*
Preventing bullying is (also) responsibility of parent.	2.92	0.38 (0.12)***	4.32	−0.21 (0.16)
2. Attitudes to parental involvement				
a. Teacher (school)				
Attitude of teacher to contact with parents in counteracting bullying.	4.78	−0.02 (0.08)	4.33	0.23 (0.09)*
Attitude of school to cooperating with parents in counteracting bullying.	4.43	0.09 (0.20)	4.02	0.35 (0.14)*
b. Parent				
Attitude of parent to cooperating with teacher in counteracting bullying.	3.95	0.26 (0.10) **	4.80	−0.03 (0.05)
Parents feel involved in creating a pleasant school atmosphere/counteracting bullying.			2.52	−0.06 (0.19)

Notes

All scales range from 1 to 5. See Appendix 1 for more information.

The clustering of respondents within schools was taken into account in the estimation of the standard errors.

*N* teachers = 89–93; *N* parents = 188–191; *N* schools = 23–27.

*p <* *0.10; **p <* *0.05; ***p <* *0.01.

**Table 2:** Primary outcomes. Efforts at the post‐assessment

	Teacher as informant		Parents of non‐victims as informant	
	Mean control	Difference intervention	Mean control	Difference intervention
1. Efforts regarding bullying				
a. Teacher				
Teacher counteracts bullying.	3.47	−0.08 (0.12)	3.30	−0.06 (0.08)
b. Parent				
Parent talks with child about school.	3.28	−0.08 (0.12)	4.58	0.03 (0.07)
How often parent talks to child about school.			3.13	0.14 (0.07) *
2. Efforts regarding parental involvement				
a. Teacher (school)				
*General efforts*				
Teacher makes parents feel welcome.	4.73	0.04 (0.11)	4.57	0.11 (0.08)
Teacher contacts parents if their child is not doing well.	4.71	−0.09 (0.12)	3.73	0.38 (0.14)*
Teacher contacts parents if their child is doing well.	3.51	0.09 (0.27)	2.96	−0.01 (0.17)
Teacher encourages parents to talk with their child about how he/she feels at school.	3.29	0.63 (0.31) *	2.64	0.36 (0.17)*
Teacher involves parents with school in general.	3.40	0.03 (0.11)	3.12	0.07 (0.05)
Teacher involves parents in counteracting bullying.	3.21	0.25 (0.14)*	3.08	0.12 (0.07)*
*Specific efforts*				
Parents are invited for individual contact with the teacher to (also) talk about how the child feels at school.	4.81	−0.11 (0.12)	4.25	0.15 (0.15)
Parents are invited for group meetings with attention to how the school tries to create a pleasant atmosphere.	3.88	0.53 (0.26)*	3.79	0.37 (0.20)*
School informs parents in writing about how the school tries to create a pleasant atmosphere.	3.94	0.46 (0.23)*	4.03	0.17 (0.16)
b. Parent				
Parents accept invitations for individual contact with the teacher.	3.02	0.06 (0.08)	3.02	0.04 (0.04)
Parents accept invitations to meetings which were also attended by other parents.	2.96	0.12 (0.13)	2.93	0.00 (0.06)
Parents read school correspondence.	3.02	0.06 (0.09)	3.14	−0.00 (0.08)

*Notes*

All scales range from 1 to 5. See Appendix 1 for more information.

The clustering of respondents within schools was taken into account in the estimation of the standard errors.

*N* teachers=84–89; *N* parents=153–191; *N* schools=23–27.

*p <* *0.10.

**Table 3:** Primary outcomes**.** Competence at the post‐assessment

	Teachers as informant		Parents of non‐victims as informant	
	Mean control	Difference intervention	Mean control	Difference intervention
1. Competence in counteracting bullying				
a. Teacher				
Competence of teacher.	3.72	0.19 (0.14)	4.00	0.15 (0.13)
b. Parent				
What parents can do to counteract bullying.	3.94	−0.04 (0.11)		
Parents learn about bullying.			2.21	0.36 (0.19)*
Parents find it difficult to counteract bullying.	2.94	−0.07 (0.11)	1.96	0.16 (0.13)
2. Competence in involving parents				
a. Teacher				
Teacher finds it difficult to involve parents in counteracting bullying.	1.85	−0.05 (0.16)		
b. Parent	n/a		n/a	

Note

All scales range from 1 to 5. See Appendix 1 for more information.

The clustering of respondents within schools was taken into account in the estimation of the standard errors.

*N* teachers=83–93; *N* parents=188–191; *N* schools=23–27.

*p <* *0.10.

### Primary outcomes: Attitudes

Table 1 shows that, at the post‐assessment, intervention teachers perceived more than control teachers did that parents disapproved of bullying (difference = 0.61, Cohen's *d *=* *0.47). Intervention teachers perceived more than control teachers did that parents felt more responsible for preventing bullying (difference = 0.38, Cohen's *d *=* *0.39). Intervention teachers felt more than control teachers did that parents placed a high value on contact with teachers to prevent and tackle bullying (difference = 0.26, Cohen's *d *=* *0.47).

The second set of columns from Table 1 provides the results for parents of non‐victimized children at post‐assessment. Contrary to our expectations, parents of non‐victimized children at intervention schools disapproved less of bullying than parents in control schools (difference = −0.35, Cohen's *d *=* *0.30). The other results for parents of non‐victimized children indicate a positive effect of the intervention on their perceptions of the teachers. Parents of the intervention group perceived that both teachers and the school considered parental contact in counteracting bullying to be of greater importance compared with control schools (differences = 0.23 and 0.35, Cohen's *d *=* *0.32, 0.35, respectively).

The findings on attitudes given in Table 1 show one unexpected effect for parents’ attitudes toward bullying, as mentioned above. Another remarkable finding is that parents and teachers at intervention schools each reported a more favorable attitude of the other informant; however, they did not report more favorable self‐reported attitudes.

### Primary outcomes: Efforts

Table 2 shows the results for the efforts to counteract bullying. The first two columns show that intervention teachers reported more often than control teachers that they encouraged parents to talk to their child about his or her feelings (difference = 0.63, Cohen's *d *=* *0.63). However, a comparable difference was found at baseline (see Appendix 3) leaving it unclear whether this effect was caused by the intervention.

Intervention teachers also reported more parental involvement in counteracting bullying (difference = 0.25, Cohen's *d *=* *0.37). Intervention teachers reported that they invited parents more frequently to school meetings about the social atmosphere (difference = 0.53, Cohen's *d *=* *0.49), and provided more written or digital information on this topic (difference = 0.46, Cohen's *d *=* *0.49) than control teachers.

The second column shows parents’ reports of efforts. Parents of non‐victims at intervention schools reported more conversations with their child about school than parents at control schools (difference = 0.14, Cohen's *d *=* *0.25). This may be a result of intervention teachers using different means to involve parents. Specifically, parents reported that intervention teachers contacted them more often when their child was not doing well (difference = 0.38, Cohen's *d *=* *0.37), encouraged them to have conversations with their children about school (difference = 0.36, Cohen's *d *=* *0.28), and involved them in counteracting bullying (difference score = 0.12, Cohen's *d *=* *0.22). Moreover, parents at intervention schools reported that they were invited more often to school meetings than parents at control schools (difference = 0.37; Cohen's *d *=* *0.31). These findings indicate that the additional efforts made by teachers at intervention schools to increase parental involvement were to some extent perceived by parents.

### Primary outcomes: Competence

Table 3 gives the results for the competence of teachers and parents in counteracting bullying (together). The first two columns show that intervention teachers did not differ from control teachers in their competence to counteract bullying or to involve parents in counteracting bullying. Parents of non‐victims in intervention schools, however reported that they had learned more about counteracting bullying than did parents of non‐victims in control schools (difference = 0.36, Cohen's *d *=* *0.31).

### Secondary outcomes

Table 4 shows the results for the secondary outcome measures: victimization and bullying, well‐being at school, and self‐esteem. There were no differences between children in intervention schools and children in control schools for any of these outcome measures, whether reported by children themselves or reported by the parents of non‐victimized children. The positive effects of the intervention on the attitudes, efforts, and competence of teachers and parents had not yet had a spillover effect on children.

**Table 4:** Secondary outcomes at the post‐assessment

	Parents of non‐victims as informant		Child as informant	
	Mean control	Difference intervention	Mean control	Difference intervention
Extent to which the child faced bullying				
How often had the child been bullied?	n/a		1.50	−0.05 (0.07)
How often had the child bullied others?	n/a		1.22	−0.06 (0.05)
Well‐being of child				
Well‐being at school.	3.46	−0.04 (0.07)	3.22	0.03 (0.05)
Self‐esteem of child				
Self‐esteem (one‐item scale). Self‐esteem.	2.83	−0.01 (0.11)	4.23	−0.01 (0.04)

*Notes*

All scales range from 1 to 5, except for self‐esteem (one‐item scale), and both scales on well‐being, which range from 1 to 4. See Appendix 2 for more information.

The clustering of respondents within schools was taken into account in the estimation of the standard errors.

Questions on bullying were not posed to parents in the post‐measure due to a mistake in the questionnaire. Victimization cannot be assessed because only parents of children who indicated being non‐victimized at t1 were included in the analyses.

*N* parents = 190‐191; *N* children = 2,510‐2,529; *N* schools = 23‐27.

## Discussion

In this study we examined the effectiveness of an intervention aimed at improving parent‐school cooperation in counteracting bullying. The effects of the intervention on the primary outcome measures of parents’ and teachers’ attitudes, efforts, and competences to tackle bullying were investigated in a randomized controlled trial with a multi‐informant approach. We found that the intervention had positive effects on some aspects of the primary outcome measures: the attitudes and efforts of both teachers and parents. There were no effects for the perceived competences of teachers. Parents, however, learned more about bullying and how to communicate with their child about bullying. Regarding the secondary outcome measures, we found no effects of the intervention on children's victimization, bullying, school well‐being, or self‐esteem. We elaborate on these findings below.

First, we expected that both teachers and parents of non‐victimized children would develop stronger anti‐bullying *attitudes* and a more positive attitude toward cooperation in counteracting bullying. We found that the intervention did have an effect on parents’ attitudes toward bullying. According to the teachers, parents at intervention schools disapproved more strongly of bullying behavior, and felt more responsible for the prevention of bullying than parents at control schools. In addition, intervention teachers reported more favorable parental attitudes to preventing and tackling bullying together than did control teachers. The parents in intervention schools compared with control schools were more positive about teachers’ attitudes toward parental involvement. Interestingly, intervention teachers were more positive about parents (but not about themselves), whereas parents were more positive about the intervention teachers (but not about themselves). Perhaps the attitudes of parents and teachers had not changed much, but had become more visible to the other informant. It is likely that teachers and parents started to communicate more often and expressed clearly their attitudes towards bullying and cooperation. Expressing their attitudes is essential because parents and teachers should be in line in order to be able to provide a clear anti‐bullying norm (Sheridan *et al*., 2004).

An unexpected finding with respect to attitudes was that parents from intervention schools disapproved less of bullying at the follow‐up compared with parents from control schools. The increased attention to bullying and its consequences at intervention schools possibly leads parents to disapprove of bullying somewhat less easily; they may realize that bullying behavior is often the result of group processes (Salmivalli, 2010) causing them to reject specific behaviors instead of rejecting the child. There may be more understanding at intervention schools for the negative behavior of children when it is caused by group processes (such as group pressure).

The other intervention effects were unequivocally positive. We expected and found a positive effect on the *efforts* of both teachers and parents of non‐victimized children in tackling bullying (together). According to both teachers and parents, parents in intervention schools were encouraged more often by the school to talk to their child, and regularly reported having conversations with their child about school. This is encouraging, as earlier research shows that many children do not tell their parents about their victimization experiences (Fekkes *et al*., 2005; Whitney & Smith, 1993). The increased frequency of the parent‐child conversations may result in a positive change. Also, teachers contributed to information sharing, because parents reported that intervention teachers made more contact if their child was not doing well. The intervention, thus, seems to facilitate the (early) signaling of bullying.

Intervention teachers reported that they provided more written or digital information about the social atmosphere at school. Furthermore, according to both teachers and parents, parents were more often invited to school meetings about the social atmosphere. These findings suggest that intervention schools increased their efforts to involve parents in counteracting bullying, which was a clear aim of the intervention.

Although we expected more *competence* of both teachers and parents of non‐victimized children in countering bullying (together), we did not find clear differences between intervention and control schools. The teachers in both intervention and control schools were motivated to tackle bullying because they were involved in the KiVa anti‐bullying program. They had been trained extensively and delivered monthly theme lessons to their students. A possible explanation for the absence of change in teachers’ competence in involving parents can be related to its difficulty; even though teachers might be good at communicating with parents, generally it is still difficult to contact them about their child being a bully or a victim. Parents of children at intervention schools, however, learned more about bullying in the past school year than parents of children at control schools. Teachers may have been able to transfer (some of their) competencies in counteracting bullying to parents.

The primary outcomes of the intervention focused on teachers and parents of non‐victims; the secondary outcomes focused on children. The results of this study were in line with our hypotheses about some aspects of the primary outcomes, but not with the hypotheses about the secondary outcomes. During the 1‐year period, the intervention had effects on the teachers and parents of non‐victims. This is a prerequisite for the actual reduction of victimization. However, it is plausible that this requires a longer time investment. Enduring effort is needed to establish clear and consistent anti‐bullying attitudes, and to increase and maintain competence in jointly counteracting bullying.

### Limitations and strengths

This study reported positive outcomes in terms of attitudes, efforts, and degree of competence in both teachers and parents. However, no effects were found for many items that tap the constructs of the primary outcome measures. Perhaps the anti‐bullying attitudes, efforts, and competences were already quite high at the baseline. Unfortunately, for some items, we have no information about the baseline because they were only included in the post‐assessment. Therefore we have no information about the starting point of the schools. Future research may include schools that do not yet apply an anti‐bullying intervention with a parental component, to investigate if the intervention makes a larger difference at these schools.

The number of respondents in the baseline and the post‐assessment differed. Fewer teachers participated in the post‐assessment, but the numbers of parents and children increased. We were not able to determine selective drop‐out. Perhaps teachers low in implementation fidelity felt less inclined to participate in the post‐assessment, whereas parents who were actively involved in the intervention probably felt more motivated to fill in the questionnaire. However, the multi‐informant approach may have led to parents providing information on non‐participating teachers and vice versa.

Despite the positive (primary) outcomes, it remains unclear whether this intervention can further increase the effectiveness of anti‐bullying programs by decreasing the bullying behavior of children. Longitudinal research with a longer time frame than the 1‐year period of the current study is needed to further investigate our encouraging findings of parents and schools as partners in counteracting bullying.

## Appendix 1 Conceptualization primary outcomes

**Table A1:** Attitudes

	Teachers as Informant	Parents of Non‐Victims as Informant
1. Attitude to bullying		
a. Teacher		
Prevention of bullying is responsibility of teacher.	It is my responsibility to prevent bullying in my class.	The teacher of my child thinks it is his/her responsibility to prevent bullying.
Attitude of school counteracting to bullying.	Everyone that works at this school thinks it is important to counteract bullying.	Everyone that works at this school thinks it is important to counteract bullying.
b. Parent		
Parent disapproves of bullying.	Most parents in my class think it is wrong to assist in bullying.	I think it is wrong if my child assists in bullying.
Preventing bullying is (also) responsibility of parent.	Most parents in my class think it is (also) their responsibility to prevent bullying in class.	*(T2)* I think it is (also) my responsibility to prevent bullying in the classroom.
2. Attitude to parental involvement		
a. Teacher (school)		
Attitude of teacher to cooperating with parents in counteracting bullying.	(α 6 items 0.77 and 0.79) I think it is important to inform parents: ‐ about how we can prevent bullying together; ‐ when I think their child is being bullied; ‐ when I think their child is bullying others. I think it is important parents inform me if they think: ‐ their child is being bullied; ‐ their child is bullying others; ‐ other children in class are being bullied.	(α 6 items 0.95 and 0.92) The teacher of my child thinks it is important to: ‐ prevent bullying together with parents; ‐ contact me when my child is being bullied; ‐ contact me when my child is bullying others. The teacher of my child thinks it is important that I initiate contact when I think: ‐ my child is being bullied; ‐ my child is bullying others; ‐ other children in class are being bullied.
Attitude of school to cooperating with parents in counteracting bullying.	Everyone that works at this school thinks it is important to work together with parents to counteract bullying.	Everyone that works at this school thinks it is important to work together with parents to counteract bullying.
b. Parent		
Attitude of parent to cooperating with teacher in counteracting bullying.	(α 5 items 0.74 and 0.67) Most parents think it is important that I contact them: ‐ about how to prevent bullying together; ‐ if I think their child is being bullied; ‐ when I think their child is bullying others. Parents from my class will inform me if they hink their child is: ‐ being bullied.; ‐ bullying others.	(α 5 items 0.67 and 0.62) I think it is important the teacher contacts me: ‐ about how to prevent bullying together; ‐ when my child is being bullied; ‐ when my child is bullying others. I think it is important to inform the teacher if I think my child is: ‐ being bullied; ‐ bullying others.
Parents feel involved in creating a pleasant school atmosphere/ counteracting bullying.		*(T2)* (α 2 items 0.88) *This school year, I felt more involved in:* ‐ *creating a pleasant atmosphere at school;* ‐ *counteracting bullying at school*.

*Notes*: Answering categories: (1) completely false; (2) somewhat true; (3) reasonably true; (4) largely true; and (5) completely true.

Questions in italics that start with ‘(T2)’ were only measured during the post measure.

Not all cells in the table are filled because not all outcome measures have been measured among from all sides.

**Table A2:** Efforts

	Teachers as informant	Parents of non‐victims as informant
1. Efforts regarding bullying		
a. Teacher		
Teacher counteracts bullying.	*(T2)* (α 3 items 0.92) *How much did you do this school year to:* * ‐ *prevent bullying;* ‐ *signal bullying;* ‐ *tackle bullying the moment it occurred?*	*(T2)* (α 3 items 0.96) *This school year, how much has the teacher done to:* * ‐ *prevent bullying;* ‐ *signal bullying;* ‐ *tackle bullying the moment it occurred?*
b. Parent		
Parent talks with child about school.	*(T2)* (α 3 items 0.89)*How often do parents from your class talk to their children about:* * ‐ *school in general;* ‐ *what the child learned at school;* ‐ *the extent to which the child feels at ease at school?*	*(T2)* (α 4 items 0.85)*I like talking to my child about:* ‐ *whom he/she plays with at school;* ‐ *what he/she learned at school;* ‐ *whether he/she likes it at school;* ‐ *whether other children are nice to him/her*.
How often parent talks to child about school.		*(T2)* *How often did you talk to your child about these topics?* *
2. Efforts regarding parental involvement		
a. Teacher (school)		
*General efforts:*		
Teacher makes parents feel welcome.	I do my best to make parents feel welcome.	I feel welcome at the school of my child.
Teacher contacts parents if their child is not doing well.	I contact parents if their child is not doing well.	School contacts me if my child is not doing well.
Teacher contacts parents if their child is doing well.	I contact parents if their child is doing well.	School contacts me if my child is doing well.
Teacher encourages parents to talk with their child about how he/she feels at school.	This school year parents were encouraged to talk with their child about how he/she feels at school.	This school year, school encouraged me to talk with my child about how he/she feels at school.
Teacher involves parents with school in general.	*(T2)*	*(T2)*
Teacher involves parents in counteracting bullying.	*How much did you do during this school year to involve parents:* * ‐ *with school in general;* ‐ *in counteracting bullying?*	*This school year, how much has the teacher done to involve parents* * ‐ *with school in general;* ‐ *in counteracting bullying?*
*Specific efforts:*		
Parents are invited for individual contact with the teacher to (also) talk about how the child feels at school.	*[The previous/this] school year parents have been invited by school to talk about their child*. During these conversations we (also) discussed whether the child likes going to school.	*[The previous/this] school year I've been invited by the school to talk about my child*. During these conversations we (also) discussed whether my child likes going to school.
Parents are invited for group meetings with attention for how the school tries to create a pleasant atmosphere.	*[The previous/this] school year, parents of my class were invited for meetings that other parents attended*. During these conversations it was (also) discussed whether their child felt at ease at school.	*[The previous/this] school year, I've been invited for meetings that other parents attended*. During these meetings attention was payed how the school tries to create a pleasant atmosphere.
School informs parents in writing about how the school tries to create a pleasant atmosphere.	[The previous/this] school year, parents from my class were informed by school in writing about the way the school tries to create a pleasant atmosphere.	[The previous/this] school year, I was informed by school in writing about the way the school tries to create a pleasant atmosphere.
b. Parent		
Parents accept invitations to individual contact with the teacher.	*(T2)* *During this school year, how often did parents accept invitations for individual appointments with you* *	*(T2)* *This school year, to what extent did you respond to invitations from school to talk about your child?* *
Parents accept invitations to meetings which were also attended by other parents.	*(T2)* *During this school year, how often did parents accept invitations for meetings that other parents attended?* *	*(T2)* *This school year, to what extent did you respond to invitations for meetings that other parents attended?* *
Parents read school correspondence	*(T2)* *How often did parents read the written information they received?* *	*(T2)* *This school year, how often did you read the writings that school send you?* *

*Notes*

Answering categories: (1) completely false; (2) somewhat true; (3) reasonably true; (4) largely true; (5) completely true

* (1) much less than the [teacher/parents of the] previous school year; (2) less than the [teacher/parents of the] previous year; (3) no difference; (4) more than the [teacher/parents of the] previous school year ‐ (5) much more than the [teacher/parents of the] previous school year.

All questions in italics that start with ‘(T2)’ were only measured during the post measure.

Not all cells in the table are filled because not all outcome measures have been measured among from all sides.

**Table A3:** Competence

	Teachers as informant	Parents of non‐victims as informant
1. Competence in counteracting bullying		
a. Teacher		
Competence teacher	(α 2 items 0.87 and 0.86) I know how to: ‐ prevent bullying; ‐ intervene when bullying occurs.	(α 2 items 0.90 and 0.92) The teacher of my child knows how to: ‐ prevent bullying; ‐ intervene when bullying occurs.
b. Parent		
What parents can do to counteract bullying.	(α 4 items 0.80 and 0.81) What can parents do:* ‐ to prevent their child from being bullied; ‐ to prevent their child from bullying others; ‐ if their child is being bullied; ‐ if their child is bullying others?	
Parents learn about bullying.		*(T2)* (α 4 items 0.94) *This school year, I learned more about:* ‐ *what bullying is;* ‐ *what the consequences of bullying are;* ‐ *how I can talk to my child about the way he/she feels at school;* ‐ *how I can talk to my child about bullying*.
Parents find it difficult to counteract bullying.	*(T2)* (α 4 items 0.89) *How difficult do the parents from your class find it to:* ** ‐ *prevent their child from being bullied;* ‐ *prevent their child from bullying others;* ‐ *intervene when their child is being bullied;* ‐ *intervene when their child is bullying others*.	(α 4 items 0.77 and 0.74) I think it is difficult to: ‐ prevent my child from being bullied; ‐ prevent my child from bullying other children; ‐ intervene when my child is being bullied; ‐ intervene when my child is bullying others.
2. Competence in involving parents		
a. Teacher		
Teacher finds it difficult to involve parents in counteracting bullying.	(α 3 items 0.78 and 0.85) I think it is difficult to: ‐ involve parents in school; ‐ contact parents if their child is being bullied; ‐ contact parents if their child is bullying others.	
b. Parent	n/a	n/a

Notes

All questions in italics that start with ‘(T2)’ were only measured during the post measure.

Not all cells in the table are filled because not all outcome measures have been measured among from all sides.

Answering categories: (1) completely false; (2) somewhat true; (3) reasonably true; (4) largely true; (5) completely true.

* (1) nothing; (2) very little; (3) a little; (4) much; (5) a great deal.

** (1) much less than the parents of the previous school year; (2) less than the parents from the class I had the previous year; (3) not much difference; (4) more than the parents of the class I had the previous year (5) much more difficult than the parents of the previous school year.

## Appendix 2 Conceptualization Secondary Outcomes

**Table A4:** Bullying, well‐being, self‐esteem

	Parents of non‐victims as informant	Child as informant
1. Extent to which the child faced bullying		
How often had the child been bullied?	Since the Summer break, how often has your child*:* ** ‐ been bullied;	Since the [Summer/Christmas] break, how often have you*:* ** ‐ been bullied;
How often had the child bullied others?	‐ bullied others.	‐ bullied others (Olweus 1996).
2. Well‐being of child		
Well‐being at school.	(α 7 items 0.94 and 0.93) My child: ‐ likes it at school; ‐ thinks it is nice and pleasant in class; ‐ feels safe when at school; ‐ likes going to school; ‐ feels accepted when at school for he/she is; ‐ feels at ease in class; ‐ is happy when at school.	(α 7 items 0.83 and 0.85) I like it at school. There is a pleasant atmosphere in my class. I feel safe at school. I like going to school. I feel being accepted as I am at school. I feel at ease in the class. I am happy when I am at school (Kärnä et al., 2011).
3. Self‐esteem of child		
Self‐esteem	My child has high self‐esteem (Robins et al., 2001).	(α 5 items 0.82 and 0.84) On the whole, I am satisfied with myself.*** I feel that I have a number of good qualities. I am able to do things as well as most other people. I feel that I am a person of worth, at least on an equal plane with others. I take a positive attitude toward myself (Rosenberg, 1965).

Note

Bullying questions were not asked to parents in the post‐measure. For that matter, victimization cannot decrease because only parents of children who at t1 were non‐victims were included in the analyses.

Legend answering categories: (1) never; (2) sometimes; (3) often; (4) always.

** (1) not; (2) one or two times; (3) two or three times a month; (4) once a week; (5) several times a week.

*** (1) never true; (2) mostly not true; (3) sometimes true; (4) mostly true; (5) always true.

## Appendix 3 Results pre‐test

**Table A5:** Primary outcomes. Attitudes at the pre‐assessment

	Teachers as informant		Parents of non‐victims as informant	
	Mean control	Difference intervention	Mean control	Difference intervention
1.Attitude to bullying				
a. Teacher				
Prevention of bullying is responsibility of teacher.	4.44	0.02 (0.11)	4.16	−0.26 (0.40)
Attitude of school to counteracting bullying.	4.87	0.07 (0.05)	4.63	0.06 (0.19)
b. Parent				
Parent disapproves of bullying.	3.83	0.20 (0.22)	4.60	0.07 (0.25)
Preventing bullying is (also) responsibility of parent.	3.03	0.05 (0.21)		
2.Attitude to parental involvement				
a. Teacher (school)				
Attitude of teacher to contact with parents in counteracting bullying.	4.71	−0.02 (0.06)	4.45	−0.02 (0.25)
Attitude of school to cooperating with parents in counteracting bullying.	4.58	−0.03 (0.09)	4.30	0.19 (0.26)
b. Parent				
Attitude of parent to cooperating with teacher in counteracting bullying.	4.01	−0.02 (0.10)	4.86	−0.01 (0.07)
Parents feel involved in creating a pleasant school atmosphere/counteracting bullying.			*(T2)*	

*Notes*

The clustering of respondents within schools was taken into account in the estimation of the standard errors *(T2)* indicates that this was only measured in the post‐test.

*N* teachers t1 = 116–121; *N* parents t1 = 94–99; *N* schools t1 = 25–27.

**p *<* *0.05; ***p *<* *0.01; ****p *<* *0.001.

**Table A6:** Primary outcomes. Efforts at the pre‐assessment

	Teachers as informant		Parents of non‐victims as informant	
	Mean control	Difference intervention	Mean control	Difference intervention
1. Efforts regarding bullying				
a. Teacher				
Teacher counteracts bullying.	*(T2)*		*(T2)*	
b. Parent				
Parent talks with child about school.	*(T2)*		*(T2)*	
How often parent talks to child about school.			*(T2)*	
2. Efforts regarding parental involvement				
a. Teacher (school)				
*General efforts:* Teacher makes parents feel welcome.	4.72	0.16 (0.08)	4.67	0.03 (0.19)
Teacher contacts parents if their child is not doing well.	4.74	0.07 (0.08)	3.73	0.37 (0.20)
Teacher contacts parents if their child is doing well.	3.84	−0.16 (0.21)	2.92	0.31 (0.29)
Teacher encourages parents to talk with their child about how he/she feels at school.	3.36	0.36 (0.23)	2.38	0.75 (0.33) *
Teacher involves parents with school in general.	*(T2)*		*(T2)*	
Teacher involves parents in counteracting bullying.	*(T2)*		*(T2)*	
*Specific efforts:* Parents are invited for individual contact with the teacher to (also) talk about how the child feels at school.	4.83	‐0.02 (0.10)	4.19	0.30 (0.23)
Parents are invited for group meetings with attention to how the school tries to create a pleasant atmosphere.	3.89	0.31 (0.32)	4.17	−0.06 (0.36)
School informs parents in writing about how the school tries to create a pleasant atmosphere.	3.77	0.12 (0.22)	3.93	0.09 (0.22)
** **b. Parent				
Parents accept invitations for individual contact with the teacher.	*(T2)*		*(T2)*	
Parents accept invitations to meetings which were also attended by other parents.	*(T2)*		*(T2)*	
Parents read school correspondence.	*(T2)*		*(T2)*	

*Notes*

The clustering of respondents within schools was taken into account in the estimation of the standard errors *(T2)* indicates that this was only measured in the post‐test.

The analyses on the pre‐test show one significant difference between control and intervention schools. This is considered trivial because a large number of tests was performed (with risk of change capitalization). However, this effect was taken into account in the interpretation of the effect at the post‐test (which is also significant).

*N* teachers t1 = 99–116; *N* parents t1 = 9; *N* schools t1 = 26–27.

**p *<* *0.05; ***p *<* *0.01; ****p *<* *0.001.

**Table A7:** Primary Outcomes. Competence at the pre‐assessment

	Teachers as informant		Parents of non‐victims as informant	
	Mean control	Difference intervention	Mean control	Difference intervention
1. Competence in counteracting bullying				
a. Teacher				
Competence of teacher.	3.66	0.03 (0.14)	3.93	0.12 (0.28)
b. Parent				
What parents can do to counteract bullying.	3.83	−0.15 (0.09)		
Parents learn about bullying.			*(T2)*	
Parents find it difficult to counteract bullying.	*(T2)*		1.91	0.08 (0.16)
2. Competence in involving parents				
a. Teacher				
Teacher finds it difficult to involve parents in counteracting bullying.	1.70	0.21 (0.10)		
b. Parent	n/a		n/a	

*Notes*

The clustering of respondents within schools was taken into account in the estimation of the standard errors *(T2)* indicates that this was only measured in the post‐test.

*N* teachers t1 = 120–121; *N* parents t1 = 94–99; *N* schools t1 = 24–27.

**p *<* *0.05; ***p *<* *0.01; ****p *<* *0.001.

**Table A8:** Secondary outcomes at the pre‐assessment

	Parents of non‐victims as informant		Child as informant	
	Mean control	Difference intervention	Mean control	Difference intervention
1. Extent to which the child faced bullying				
How often had the child been bullied?	n/a		1.61	–0.13 (0.07)
How often had the child bullied others?	1.05	0.03 (0.04)	1.23	–0.03 (0.04)
2. Well‐being of child				
Well‐being at school.	3.50	0.03 (0.10)	3.26	0.01 (0.05)
3. Self‐esteem of child				
Self‐esteem (1 item scale)	2.92	–0.12 (0.16)		
Self‐esteem.			4.20	0.02 (0.05)

*Notes*

The clustering of respondents within schools was taken into account in the estimation of the standard errors *(T2)* indicates that this was only measured in the post‐test.

Only parents of non‐victimized children were included therefore ‘How often had your child been bullied?’ has no value.

*N* parents t1=98–99; *N* children t1=2,446–2,447; *N* schools t1=25–27.

*<0.05;***p*<0.01; ****p*<0.001.
